# The role of exhausted natural killer cells in the immunopathogenesis and treatment of leukemia

**DOI:** 10.1186/s12964-023-01428-2

**Published:** 2024-01-22

**Authors:** Asal Barshidi, Keivan Ardeshiri, Farbod Ebrahimi, Fatemeh Alian, Ali Akbar Shekarchi, Mohammad Hojjat-Farsangi, Farhad Jadidi-Niaragh

**Affiliations:** 1 Department of Biological Sciences, Faculty of Sciences, University of Kurdistan, Sanandaj, Iran; 2 Department of Biology, Science and Research Branch, Islamic Azad University, Tehran, Iran; 3 Nanoparticle Process Technology, Faculty of Engineering, University of Duisburg-Essen, Duisburg, Germany; 4 Institute of Biochemistry and Biophysics, University of Tehran, Tehran, Iran; 5 Department of Pathology, Faculty of Medicine, Tabriz University of Medical Sciences, Tabriz, Iran; 6 Bioclinicum, Department of Oncology-Pathology, Karolinska Institute, Stockholm, Sweden; 7 Immunology Research Center, Tabriz University of Medical Sciences, Tabriz, Iran; 8 Department of Immunology, Faculty of Medicine, Tabriz University of Medical Sciences, Tabriz, Iran; 9 Research Center for Integrative Medicine in Aging, Aging Research Institute, Tabriz University of Medical Sciences, Tabriz, Iran

**Keywords:** Natural killer cells, Leukemia, Cancer, Exhaustion, Hematopoietic malignancies

## Abstract

The immune responses to cancer cells involve both innate and acquired immune cells. In the meantime, the most attention has been drawn to the adaptive immune cells, especially T cells, while, it is now well known that the innate immune cells, especially natural killer (NK) cells, play a vital role in defending against malignancies. While the immune cells are trying to eliminate malignant cells, cancer cells try to prevent the function of these cells and suppress immune responses. The suppression of NK cells in various cancers can lead to the induction of an exhausted phenotype in NK cells, which will impair their function. Recent studies have shown that the occurrence of this phenotype in various types of leukemic malignancies can affect the prognosis of the disease, and targeting these cells may be considered a new immunotherapy method in the treatment of leukemia. Therefore, a detailed study of exhausted NK cells in leukemic diseases can help both to understand the mechanisms of leukemia progression and to design new treatment methods by creating a deeper understanding of these cells. Here, we will comprehensively review the immunobiology of exhausted NK cells and their role in various leukemic malignancies.

Video Abstract

**Supplementary Information:**

The online version contains supplementary material available at 10.1186/s12964-023-01428-2.

## Introduction

Regarding the increased prevalence of hematopoietic malignancies and the existence of difficulties in treatment, it is essential to study the etiology and immunopathogenesis of blood cancers, especially leukemias. Despite all the progress achieved, chemotherapy is the main therapeutic strategy for almost all hematopoietic malignancies which magnifies the importance of identifying novel and efficacious therapeutic targets [1, 2].

Both innate and adaptive immune responses are critical in the defense against cancer cells. Although it is generally supposed that adaptive immune cells, particularly T cells, are an essential part of the immune response against cancer cells, natural killer (NK) cells also play a critical role in the defense against malignant cells. They are the most critical innate immune lymphocytes in defense against infections and cancers. While impaired cytotoxic function of NK cells is correlated with cancer progression, the upregulation of activating receptors on NK cells is correlated with better disease prognosis [1, 2]. Similarly, the accumulation of functional NK cells in the tumor microenvironment has also been associated with low grades of cancer [3, 4].

Cancer cells can induce exhaustion in NK cells by changing the phenotype and function of NK cells and suppressing their anti-tumor function. The term "exhaustion" was initially used for T lymphocytes, wherein these cells undergo phenotypic changes and functional impairment following repeated exposure to antigens under pathological conditions, such as cancer or chronic infection. Induction of this state is associated with extensive alterations in T lymphocytes, including the induction of the inhibitory immune checkpoints, metabolic changes, epigenetic changes, and changes in molecular signaling pathways [5]. It should be noted that the exhaustion process and features are different in T and NK lymphocytes, which may be due to the fundamental differences between these two cells. While T cells have very diverse antigen receptors and identify antigens depending on MHC molecules [6, 7].

The evidence indicates that the occurrence of exhausted NK cells in leukemic patients has significantly increased, and their anti-leukemic activity has been dramatically inhibited [8, 9]. On the other hand, leukemia treatments based on targeting these cells have been associated with promising results, which indicate the introduction of a new treatment method. It seems necessary to mention that using NK cells in treating hematopoietic malignancies has been more successful than solid tumors. This issue is probably due to the difficulty of infiltrating NK cells to the tumor site and exposure to inhibitory signals in the tumor microenvironment, suppressing NK cell activity and inducing their exhaustion [10].

Despite efforts, many ambiguous and unknown issues in this field still require detailed and comprehensive studies. In this review article, we will try to discuss the immunobiology of NK cells, exhaustion of NK cells, the role of exhausted NK cells in leukemia, and targeting these cells for the treatment of leukemia.

## Immunobiology of NK cells

NK cells are the most important components of the innate immune system that originate from the bone marrow and are present in the bloodstream and tissues such as lymph nodes, liver, thymus, and uterus [11].

Different subtypes of NK cells have been identified in humans and mice. The expression of molecules such as NK.1, CD49b, and NKp46 determine NK cells in mice. In humans, the CD16^+^CD56^+^ phenotype represents these cells [12]. This classification is partly derived from the differentiation stages of these cells. Accordingly, four stages of differentiation have been observed in murine NK cells. Primary immature NK cells have a CD27^−^CD11b^−^ phenotype. In the second stage, these cells obtain the expression of molecules such as CD27, NK1.1, NKp46, and NKGD2. The third step starts with the expression of CD27, CD11b, and S1P5 molecules on the cell surface. In the fourth stage, mature cells will have a CD27^−^CD11b^+^KLRG1^+^ phenotype that exhibits cytotoxic function.

On the other hand, the differentiation of NK cells in humans includes five stages. In the first stage in the bone marrow, pre-NK cells originate from lymphoid progenitors. In the second stage, these cells express the IL-15 receptor to ensure their survival during this stage until stage four. CD3^−^CD56^bright^CD16^−^ cells are the product of the fourth stage of differentiation, which resides mainly in the lymph nodes, produces a high amount of cytokines, and has low toxicity. The outcome of the fifth stage of differentiation is CD3^−^CD56^dim^CD16^+^ cells, which are mainly present in blood circulation or inflammatory tissues. These cells exert cytotoxic function through the production of perforin and interferon (IFN)-gamma [13, 14]. 

According to another classification, within CD56^dim^ cells, there are two types, including conventional NK cells and adaptive NK cells [15]. Adaptive NK cells have a different metabolic profile and epigenetic characteristics similar to effector CD8^+^ T cells [16]. These cells are long-lived; many express CD94/NKG2C [17]. These cells, mainly identified in mice, can survive over six months and self-renew. So far, three categories of adaptive NK cells have been identified, including hepatic-liver-resident NK, cytokine-induced memory-like NK, and cytomegalovirus (CMV)-specific NK (Specific NK cells against CMV) [18]. Adaptive NK cells exhibit antigenic specificity levels and memory recall properties. While the lifespan of conventional NK cells is less than ten days, adaptive NK cells can survive for months and even years [19].

Functionally, NK cells have a high ability to identify and kill virus-infected cells and transformed cells. These cells do not need prior exposure to the antigen to recognize the target and identify antigens through germline-encoded receptors. NK cells express various inhibitory and activating receptors, whose interaction with different ligands leads to activating or inhibiting their activity following target cell recognition. It is essential to mention that in the case of the simultaneous connection of activating and inhibitory receptors with their ligands, inhibitory signals will dominate, probably to maintain homeostasis and prevent self-directed responses [20]. NK cells express several inhibitory receptors, including T-cell immunoreceptor with immunoglobulin and immunoreceptor tyrosine-based inhibitory motif domains (TIGIT), programmed death protein 1 (PD-1), T-cell immunoglobulin and mucin domain-containing protein 3 (TIM3), CD96, CD112R, interleukin (IL)-1R8, NKG2A, killer-cell immunoglobulin-like receptors (KIRs), and lymphocyte-activation gene 3 (LAG-3). The activating receptors of these cells also include NKG2, NKG2D, and CD226 [21]. NK cells express many inhibitory checkpoints of T cells, which led many researchers to think that it is possible to enhance the activity of NK cells by inhibiting these checkpoints and preventing exhaustion, similar to what has been experienced with T lymphocytes.

NK cells use a variety of mechanisms to activate and kill virus-infected or transformed cells. Identifying cells that lack MHC I molecules can lead to the non-activation of inhibitory checkpoints and, as a result, the dominance of NK cell activating receptors.Antibody-dependent cell-mediated cytotoxicity (ADCC) is another important mechanism of NK cell activation, in which NK cells bind to antibody-mediated opsonized particles through CD16 receptor. Moreover, some inflammatory cytokines can also activate NK cells [22, 23].

## NK cell exhaustion

**Fig. 1:**
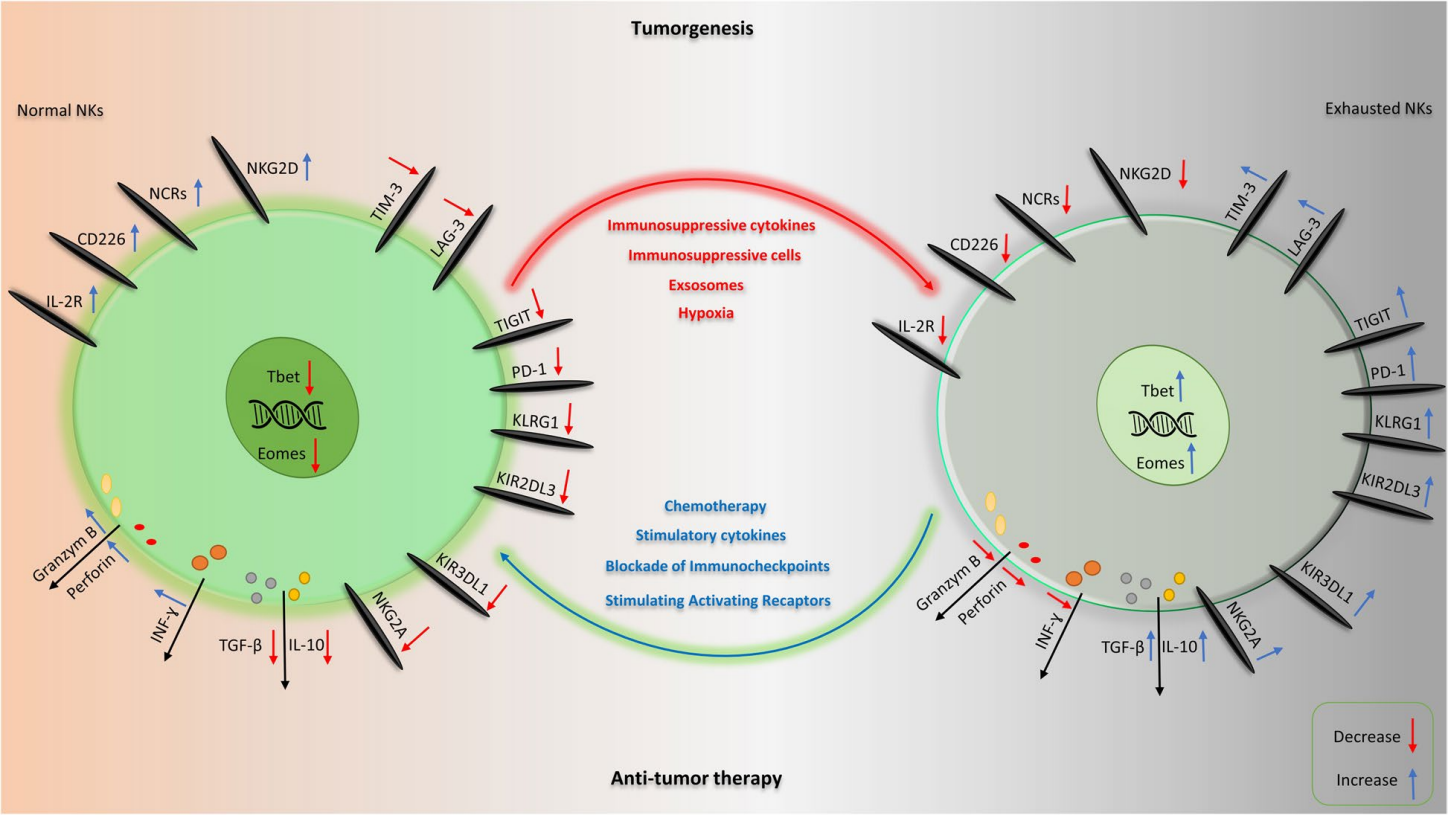
Phenotypic and functional properties of NK cells in cancer. While immunosuppressive cells or cytokines, exosomes, and hypoxia promote exhaustion of NK cells, chemotherapy, stimulatory cytokines, blockade of immune checkpoint molecules, and stimulating activating receptors prevent exhaustion

Exhaustion of immune cells is a state in which immune cells are defective regarding function and proliferative capacity. Exhaustion associated with a significant transcriptional profile alteration which is a consequence of extensive phenotypic, metabolic, and epigenetic alterations. Exhaustion primarily transpires in the presence of antigens and through recurrent stimulation, exemplified by chronic infections and malignancies. Considering the lifespan of NK cells, it seems that the exhaustion is mainly related to adaptive NK cells, which have long-term survival and can be chronically and repeatedly exposed to infectious or tumor antigens. However, there is evidence indicating that conventional natural killer (NK) cells can also experience an exhaustion process following repeated stimulation with cytokines or infectious agents [24]. As mentioned earlier, the exhaustion of NK cells is associated with various phenotypic, metabolic, functional, and epigenetic changes, which we describe below (as shown in Fig. 1).

The augmentation of the expression of inhibitory receptors serves as a critical marker for the state of exhaustion in natural killer cells. Accordingly, increased expression of the PD-1 molecule is considered one of the most critical indicators of exhausted cells in both T and NK cells. The PD-1 molecule is not only an indicator of exhausted NK cells, but its signaling plays a significant role in the exhaustion process [25]. The upregulation of PD-1 in CD56^dim^NKG2A^−^KIR^+^CD57^+^ NK cells has been observed in both solid tumors and hematopoietic malignancies. These elevated PD-1 levels were accompanied by reduced cytokine secretion, defective degranulation, and decreased proliferation [1, 26, 27].

It should be noted that some studies have suggested that the expression of TIGIT and, to a lesser extent, LAG-3 and TIM-3 are more critical compared to PD-1 as an indicator of NK cell exhaustion [28, 29]. Another molecule whose signaling is mentioned as one of the influential factors in inducing NK exhaustion is NKG2A. CD56^dim^ NKG2A^high^ NK cells were also increased in hepatocellular carcinoma patients, which was associated with a poor prognosis [30]. KIR receptors family and CD96 are other inhibitory molecules increased in exhausted NK cells.

Moreover, the decrease in the expression of stimulatory receptors is correlated with the exhaustion of NK cells. There has been a regular observation of the diminished presence of the stimulatory receptor NKG2D in different types of solid tumors or leukemia [31]. The reduced expression of NKG2D was also correlated with the decreased DAP10 expression [32]. Furthermore, decreased expression of several other NK cell activating receptors, including NKp30, NKp44, NKp46, CD16, 2B4, and CD226, has also been demonstrated in cancer patients, associated with poor prognosis [1]. Given that the equilibrium between the signals obtained from the inhibitory and activating receptors of NK cells will ultimately dictate the outcome of cellular activity, it appears that the decrease in expression levels of activating receptors in individuals with cancer results in the promotion of inhibitory signals prevailing in these cells.

From a functional point of view, it has been determined that NK cells have an incomplete function in a tumor environment or chronic infection and lose the ability to perform cytotoxic activities. For example, cancer progression in murine models is associated with the decreased frequency and function of NK cells [33, 34]. Furthermore, adoptively transferred NK cells to leukemic mice also lost their cytotoxic ability after exposure to malignant cells [22]. Also, tumor-infiltrating NK cells exhibit impaired cytotoxic functions, which is in part related to the downregulation of IFN-γ, FasL, perforin, CD107a, granzyme B, and tumor necrosis factor-related apoptosis-inducing ligand (TRAIL) [30, 31]. 

 In addition to phenotypic and functional modifications, there seem to be some transcription profile changes in exhausted NK cells in the tumor area. It has been reported that transcription factors T-bet and eomesodermin (Eomes), which play an essential role in NK cells' activity, differentiation, and maturation, are significantly reduced in exhausted NK cells [22, 35]. Adoptively transferred NK cells to the murine leukemia model exhibited the reduced expression of these transcription factors, which was associated with impaired NK cell function. Interestingly, the induction of Eomes expression in these cells partially restored the function of NK cells [22]. This finding shows that the reduction of Eomes indicates exhaustion in NK cells and part of its induction process.

To understand the induction of NK cell exhaustion, it is necessary to examine the influential factors implicated in this process. One of the influential factors in NK exhaustion is the immunosuppressive microenvironment of the tumor. The most important immunosuppressive cells include regulatory T cells (Tregs) [36], myeloid-derived suppressor cells (MDSCs) [37], tumor-associated fibroblasts [38], tumor-associated neutrophils [39], and tumor-associated macrophages [40]. The upregulation of immunosuppressive cells in cancer patients is associated with decreased frequency and function of NK cells. These inhibitory cells can inhibit NK cells and induce exhaustion with various mechanisms dependent on cell–cell contact or independent of cell attachment (through secretion of immunosuppressive factors) [41–43]. Immune inhibitory cytokines such as transforming growth factor-β (TGF-β) and IL-10 are also essential in NK cell exhaustion [44, 45]. The hypoxic condition of the tumor microenvironment is an important factor in the suppression and exhaustion of NK cells. The hypoxia downregulates NKG2D, NKp46, NKp30, and NKp44 in NK cells through the expression of hypoxia-inducible factor 1α (HIF-1α) [46, 47].

The upregulation or downregulation of certain factors on cancer cells, which act as ligands for inhibitory or activating receptors of NK cells, is considered to be one of the most crucial elements that lead to NK cell exhaustion. As mentioned, the balance between the signals received from activating and inhibitory receptors of NK cells will determine their function. This balance will be towards inhibitory signals in the vicinity of malignant cells. Numerous investigations have demonstrated the notable presence of diverse molecules on the surface of malignant cells, which serve as the ligands for the suppressive receptors of NK cells. Consequently, this phenomenon has resulted in the impairment of NK cells and, ultimately, their exhaustion. For example, the increased expression of CD200 and galectin-9 on cancer cells (ligands of CD200R and TIM-3 on NK cells, respectively) has been demonstrated in acute myeloid leukemia (AML) [48, 49]. On the other hand, the downregulation of CD48 on leukemic cells (the ligand for activating receptor 2B4 on NK cells, was observed in AML [50].

Finally, exosomes secreted from cancer cells are also effective factors in the inhibition and exhaustion of NK cells. Exosomes with different mechanisms such as the secretion of inhibitory cytokines such as TGF-β, providing microRNAs involved in NK suppression or the expression of inhibitory receptor ligands of NK cells can lead to defects in the cytotoxic activity of these cells and facilitate the exhaustion process [51, 52]. 

## Exhausted NK cells and leukemia

The inactivation of NK cells and their failure to eradicate leukemic cells is a prevailing occurrence noted in nearly all leukemic malignancies. In this section, we will review the studies on the inefficiency of NK cells in various leukemias, the mechanisms of NK exhaustion, and the therapeutic strategies used to strengthen NK cells.

### CLL

The ability of ammonium chloride to inhibit the cytotoxic activity of NK cells cultured with the leukemia cell line K562 (chronic myelogenous leukemia) was one of the first experiences related to the inhibition of these cells in leukemia. This suppression was reversible (after 15 h) and dose-dependent [53].

One of the most common ways to study exhausted NK cells is to examine their frequency in patients and their correlation with disease prognosis. Accordingly, an increased frequency of exhausted NK cells has been reported in chronic lymphocytic leukemia (CLL) patients. By studying the peripheral blood of 24 CLL patients and 19 normal individuals, Hadadi et al*.* have shown that the frequency of CD56^+^CD3^−^Tim-3^+^ cells has increased significantly in these patients, which was associated with the downregulation of CD56^+^CD3^−^NKp30^+^ cells. They indicated that these dysregulated frequencies were correlated with poor prognostic factors such as high absolute lymphocyte count, decreased hemoglobin, and elevated serum C reactive protein (CRP) concentration [54].

Some studies have shown the effect of some common treatments on NK cell exhaustion in leukemia patients. For example, the anti-CD20 monoclonal (ofatumumab or rituximab) antibody could promote NK cell exhaustion by binding to CD16 on NK cells. This interaction could impair the cytotoxic activity of NK cells. Following this interaction, some NK cells activating receptors such as NKp46, NKG2D, 2B4, and DNAM-1 could not phosphorylate essential signaling molecules involved in the cytotoxic function of NK cells, such as phospholipase C (PLC)γ2, SH2-domain-containing leukocyte protein of 76 kDa (SLP-76), and Vav1. Furthermore, this ligation could also recruit the inhibitory phosphatase Src homology region 2 domain-containing phosphatase-1 (SHP-1) to the cytoplasmic tail of CD16, leading to further suppression of NK cells. These findings interestingly show the dual role of the CD16 receptor in the activation or inhibition of NK cells and provide a new mechanism for the exhaustion of these cells because the pharmacological blockade of this receptor led to the recovery of the cytotoxic activity following binding to anti-CD20 antibody [55]. Although these findings imply that rituximab can inhibit some anti-leukemia responses, it should be evaluated in further studies to evaluate its advantages and disadvantages.

On the contrary, there exists information suggesting that certain therapies can impede the exhaustion of NK cells and enhance the cytotoxic capabilities of these cells. Accordingly, NK cells derived from healthy subjects after treatment with drugs such as sunitinib, sorafenib, or the pan-RAF inhibitor ZM336372 had a high ability to secrete cytokines and kill target cells and prevent exhaustion in the RAS/RAF/ERK signaling pathway-dependent manner [56]. Similarly, treatment of 55 relapsed/refractory and 50 untreated CLL patients with Ibrutinib was associated with increased effective NK cells compared to 20 normal subjects [57]. Likewise, during four years of follow-up of 31 CLL patients and 20 normal subjects, Ibrutinib could enhance the frequency and function of NK cells, which was associated with good prognosis [58].

The expression of checkpoints on NK cells and their engagement with the corresponding receptors on leukemic cells stands as a highly crucial means by which NK cells experience suppression and exhaustion. Likewise, the endeavor to target inhibitory checkpoints on NK cells has been recognized as a preeminent immunotherapeutic approach for targeting exhausted NK cells. The study of circulating NK cells in 17 CLL patients showed that leukemic B cells express significantly higher levels of Siglec-7 ligands than normal individuals, which was associated with poor disease prognosis. Moreover, the blockade of the Siglec-7 ligand markedly enhanced the sensitivity of leukemic cells to the cytotoxic effects of NK cells [59]. In another study, the expression of LAG3 was significantly increased in leukemic and NK cells of 61 untreated CLL patients, which was associated with a poor prognosis. In addition, the blockade of LAG3 using monoclonal antibodies significantly preserved the cytotoxic function of NK cells against leukemic cells [60]. B and T lymphocyte attenuator (BTLA) is another inhibitory checkpoint its upregulation is detected on both leukemic and NK cells in 46 untreated CLL patients associated with a poor prognosis. Interestingly, ex vivo blockade of BTLA led to the depletion of leukemic cells and enhanced cytotoxic function and cytokine secretion by NK cells [61]. Human inhibitory receptors Ig-like transcript 2 (ILT2) (also known as LIR-1 or LILRB1) is also an immune checkpoint investigated in CLL patients regarding its role in suppressing NK cells. While B-CLL cells exhibited low levels of this ILT2, it was upregulated in the NK cells of CLL patients (*n* = 60) compared to normal individuals (*n* = 25), which was associated with the severity of disease. It has been reported that inhibiting ILT2 with Lenalidomide significantly activates NK cells and eliminates leukemic cells [62]. In contrast, blockade of PD-1 and TIM-3 checkpoints in NK cells derived from the peripheral blood of 18 early-stage CLL patients did not affect the cytotoxic function and secretion of tumor necrosis factor (TNF)-α and IFN-γ [63].

As we reviewed in this section (summarized in Table 1), despite the interesting clues regarding the role of exhausted NK cells in CLL patients, there are still many unknowns in this field. In future studies, different subtypes of exhausted NK cells should be investigated in patients with varying degrees of disease progression. Future investigations should comprehensively address the differences between peripheral blood and bone marrow NK cells. Also, many other checkpoints have not yet been investigated in CLL patients, which should be comprehensively evaluated. Furthermore, the efficacy of targeting exhausted NK cells in CLL patients as a therapeutic target should be precisely investigated in preclinical and clinical studies.

**Table 1:** Studies related to the role of exhausted NK cells in CLL

Main findings	Ref
Ammonium chloride can inhibit the cytotoxic activity of NK cells in vitro	[53]
The upregulation of circulating CD56^+^CD3^−^Tim-3^+^ exhausted NK cells in CLL patients was associated with the upregulation of CD56^+^CD3^−^Tim-3^+^ NK cells associated with disease progression	[54]
Anti-CD20 monoclonal antibodies ofatumumab or rituximab) can cause the exhaustion of NK cells in CLL patients through binding to CD16 on NK cells	[52]
Treatment of 55 relapsed/refractory and 50 untreated CLL patients with Ibrutinib was associated with increased effective NK cells compared to 20 normal subjects	[57]
The long-term treatment of CLL patients with Ibrutinib increased NK cells in peripheral blood, which was associated with a good prognosis	[58]
Leukemic B cells of CLL patients express significantly higher levels of Siglec-7 ligands than normal individuals, which was associated with poor disease prognosis	[59]
The expression of LAG3 was significantly increased in leukemic cells and NK cells and was associated with a poor prognosis Inhibiting this checkpoint increased the cytotoxic activity of NK cells against leukemic cells	[60]
The upregulation of BTLA is detected in both leukemic cells and NK cells in untreated CLL patients, which was associated with a poor prognosis Ex vivo blockade of BTLA led to the depletion of leukemic cells and enhanced cytotoxic function and cytokine secretion by NK cells	[61]
The expression of ILT2 was increased in the NK cells of CLL patients (*n* = 60), which was associated with a poor prognosis Inhibiting ILT2 with Lenalidomide significantly activated NK cells and eliminated leukemic cells	[62]
Inhibition of PD-1 and TIM-3 receptors in circulating NK cells of 18 early-stage CLL patients did not affect the recovery of cytotoxic function and secretion of TNF-α and IFN-γ	[63]

### AML

Among the various malignancies of leukemia, the majority of research regarding the significance of exhausted NK cells has been conducted in patients with AML. A particular investigation within this context investigated the impact of genetic polymorphism in the coding sequences of receptors responsible for the inhibition or activation of NK cells, and its potential association with the susceptibility to AML. While AML patients (*n* = 169) exhibit high expression of KIR activating receptors, especially KIR3DS1, normal subjects (*n* = 167) express KIR inhibitory receptors, especially KIR2DL1 and KIR3DL1. It has been proposed that the increased expression of KIR-activating receptors in patients probably leads to the hyperactivation of these cells following the encounter with leukemic cells and further the exhaustion of NK cells and the progression of cancer [64]. Interestingly, some fungal infections, such as A. *fumigatus,* promote NK cell exhaustion in AML patients leading to their reduced cytotoxic function against leukemic cells. Exhaustion was associated with the reduced secretion of inflammatory cytokines such as TNF-α, IFN-γ, regulated upon activation, normal T cell expressed and presumably secreted (RANTES), macrophage inflammatory protein (MIP)-1α, and MIP-1β, decreased expression of activating receptors such as NKG2D and NKp46, and impaired NK cell degranulation [65]. Therefore, some infections in leukemic patients can also be considered one of the causes of NK cell exhaustion.

The frequency and absolute number of peripheral CD56^+^CD16^+^ NK cells were also markedly decreased in myelodysplastic syndromes (MDS) and AML secondary to MDS patients (*n* = 130) compared to normal subjects (*n* = 40). It was also shown that NK cells expressing NKG2D, NKp46, and CD161 were significantly reduced in patients compared to normal subjects [66]. Zeng and colleagues also proposed that NK cells in the peripheral blood of AML patients are in an exhaustion state. In contrast, NK cells in the bone marrow mainly have a terminally differentiated phenotype, which correlates with low patient survival. They showed that the frequency of CD16^−^CD56^dim^ NK cells is reduced in the peripheral blood of novo AML and AML patients with complete remission after chemotherapy, whereas there was no change in the bone marrow. In addition, while the frequency of killer cell lectin-like receptor G1 (KLRG1)- and TIGIT-expressing NK cells was increased in peripheral blood of novo patients, it was recovered in patients after chemotherapy. Moreover, the frequency of terminally differentiated NK cells (CD56^dim^CD16^+^CD57^+^), which had a potent cytotoxic function and low replication capacity, was increased in the bone marrow of novo AML patients and correlated with a lower survival rate [67]. Tang et al*. also* demonstrated that peripheral NK cells of AML patients (*n* = 79) had extensive functional impairments, which were correlated with disease relapse and resistance to treatment. Also, in patients who responded to chemotherapy, the functional responses of NK cells were restored [68]. By studying the bone marrow samples of 37 newly diagnosed AML patients, it was found that the expression of TIM-3 checkpoint on NK cells and blasts can be used as a prognosis marker [69]. The review of these three studies shows that the exhaustion status of NK cells in AML patients with various degrees of progression is different. Another point that can be taken from these studies is the difference between exhausted NK cells in peripheral blood and bone marrow. Finally, it seems that the exhaustion status in NK cells is reversible, and after treatment with drugs such as chemotherapy, the activity of these cells returns to normal conditions.

Bou-Tayeh and coworkers demonstrated that the deregulation of signaling pathways induced by cytokines in NK cells of AML patients might constitute one of the mechanisms implicated in the exhaustion process. They provided evidence that the advancement of AML correlated with impairments in the maturation and functionality of NK cells in murine models of AML. While the IL-15/mammalian target of rapamycin (mTOR) and type I interferon (IFN) signaling pathways were constitutively active in NK cells purified from the leukemia murine model, these cells could not respond to stimulation with IL-15, in vitro. Therefore, chronic activation of NK cells through the IL-15/mTOR pathway is one of the NK cell exhaustion mechanisms in AML models. Similar results were observed in NK cells derived from AML patients in vitro. There were low expression levels of IL-2/15Rβ and decreased response to stimulation with IL-15 in these cells [70].

Targeting checkpoint molecules has been introduced as an effective immunotherapy method for targeting exhausted NK cells. Recently, poliovirus receptor-related immunoglobulin domain-containing (PVRIG) has been introduced as one of the new effective checkpoints inhibiting NK cells' activity, especially CD56^bright^ cells. By studying the blasts of 20 AML patients, it has been shown that these cells continuously express the ligand of this checkpoint, poliovirus receptor-related 2, PVRL2. Interestingly, blockade of the PVRIG/PVRL2 axis using an anti-PVRIG antibody markedly activated NK cells to kill PVRL2-expressing leukemic cells. In contrast to peripheral NK cells, bone marrow NK cells had no upregulated PVRIG expression. Activation of NK cells following ligation of activating receptors (NKp46 and CD16), leukemic cell recognition, or cytokine stimulation (IL-12 and IL-2) could suppress the expression of PVRIG in NK cells [71].

PD-1/PD-L1 axis is another checkpoint that was well investigated in various cancers. An exciting recently published finding showed that NK cells in contact with leukemic cells in the C1498 murine AML model acquire the PD-1 molecule from leukemic cells through the Trogocytosis mechanism in a SLAM receptor-mediated manner. Trogocytosis denotes a biological phenomenon characterized by the physical extraction and ingestion of cellular material, referred to as "bites," from one cell by another cell [72]. Therefore, PD-1 checkpoint expression on NK cells seems intrinsic and can originate from leukemic cells.

Moreover, exhausted NK cells are worthy targets for the treatment of PD-L1^−^ tumors. Dong and coworkers showed that the ameliorative effect of anti-PD-L1 antibody in PD-L1^−^ AML (*n* = 79) is related in part to the targeting of PD-L1^+^ exhausted NK cells. Malignant cells induce PD-L1 in NK cells through the AKT signaling pathway. The impact of anti-PD-L1 antibody on NK cells is also through the P38 pathway. Furthermore, the combination use of anti-PD-L1 antibody and NK cell-stimulating cytokines had a more significant impact on leukemic cells compared to monotherapy [73]. These findings imply that the rationale for using an anti-PD-L1 antibody in PD-L1 negative tumors can be targeting exhausted NK cells. The mouse model of AML has yielded comparable findings with regard to the inhibition of PD-L1, underscoring the significance of checkpoint inhibition in averting NK cell exhaustion and enhancing their efficacy against leukemia [74].

Evaluation of NK cells in peripheral blood of 100 AML patients showed increased expression of B7-H3 checkpoint, which was associated with poor prognosis. Also, inhibition of this checkpoint using monoclonal antibody had a significant impact on the cytotoxic function of NK cells, both in vitro (in HL-60, Kasumi-1, THP-1, MV4-11, MOLM-13, U937, OCI-AML3, OCI-AML2, and MOLM-14 cells) and in vivo. Treatment of AML patient-derived xenografts with an anti-B7-H3 antibody had similar results [75].

Another checkpoint that plays a role in inhibiting the function of NK cells is TIGIT, which binds to its ligands, including CD155 and CD112, on leukemic cells. In an in vitro study conducted on AML cell lines (MOLM-13, MV-4–11, NB-4, THP-1, and KG-1), inhibition of CD155/CD122 on leukemic cells by Flt3 inhibitors enhanced the cytotoxic function of NK cells against these cells [76]. TIGIT was also upregulated on NK cells and bone marrow samples in AML patients following allogeneic transplantation, which was associated with the downregulation of NK cells in the bone marrow [77]. Likewise, blockade of TIGIT, CD39, and A2AR checkpoints on NK cells purified from peripheral blood (*n* = 15) and bone marrow (*n* = 25) of AML patients increased the cytotoxic function of NK cells [78].

Furthermore, the examination of TIM-3 expression in NK cells in 47 newly diagnosed AML patients showed that the expression of this checkpoint is associated with a poor prognosis and has prognostic significance [79]. In a similar study, newly diagnosed AML patients (*n* = 23) had a higher frequency of TIGIT^+^PD-1^+^TIM3^+^ NK cells compared to normal subjects, which was associated with poor prognosis. The expression of these checkpoints was associated with low cytotoxicity, and their inhibition increased the function of NK cells [80]. In contrast, in a study on 150 AML patients, Rakova and coworkers reported that TIM-3 expression in NK cells correlates with high functional capacity and better clinical outcomes. Moreover, in contrast to M1 and M2 patients, M4 and M5 patients had a lower frequency of NK cells compared to normal subjects. They also showed intact TIGIT and significant downregulation of PD-1 in patients-derived NK cells compared to healthy donors. Intriguingly, the blockade of TIM-3 (but not PD-1 and TIGIT) inhibited the secretion of IFN-γ in NK cells following stimulation [81].

The use of anti-NKG2A antibodies in the mouse model of AML led also to significant activation of NK cells and tumor regression [82].

In addition to the inhibitory receptors, investigating the activating receptors of NK cells can also be considered a solution to prevent the exhaustion of these cells and increase their anti-leukemic activity. Accordingly, by studying 111 AML patients, it has been determined that the interaction of the OX40 molecule on leukemic cells with the OX40L checkpoint causes the activation of NK cells and eliminates leukemic cells [83].

The design and production of CAR-NK cells is another advanced and innovative method of treating leukemia. Gurney et al*.,* using transposon-engineered CAR-NK cells, could efficiently target and destroy AML cell lines with expression of CLL-1 molecule and primary AML cells, even leukemia stem cells. They further engineered CAR-NK cells by silencing NK cell cytokine checkpoint cytokine-inducible SH2-containing protein (CIS) using the CRISPR/Cas9 system, which was associated with increased cytotoxicity of these cells [84].

Taken together, it seems that the progression of AML is associated with an increase in the frequency of exhausted NK cells, accompanied by a high expression of inhibitory checkpoints and a decrease in the expression of activating receptors and functional defects in these cells (Table 2). Also, although the results of the studies are not sufficient and comprehensive, it seems that exhausted NK cells in the peripheral blood and bone marrow of AML patients do not have similar conditions; however, this field should be studied comprehensively. The results of the studies that have used the inhibition of NK cell checkpoints for treating AML are promising. Still, they should be evaluated in more detail in mouse models and clinical efficacy.

**Table 2:** Studies related to the role of exhausted NK cells in AML

Main findings	Ref
The increased expression of KIR activating receptors in ALL patients leads to the hyperactivation of these cells following the encounter with leukemic cells and further the exhaustion of NK cells and the progression of cancer	[64]
Some fungal infections, such as A. *fumigatus,* cause exhaustion in NK cells derived from AML patients and significantly inhibit their cytotoxic activity against leukemic cells	[65]
The frequency and absolute number of CD56^+^CD16^+^ NK cells in the peripheral blood of AML patients significantly decreased compared to normal subjects NK cells expressing NKG2D, NKp46, and CD161 were significantly reduced in patients compared to normal subjects	[66]
NK cells in the peripheral blood of AML patients are in an exhaustion state NK cells in the bone marrow mainly have a terminally differentiated phenotype, which correlates with low patient survival	[67]
Circulating NK cells in AML patients had many functional defects associated with excessive maturation and significant reduction of NKG2D and NKp30 expression In patients who responded to chemotherapy, the functional responses of NK cells were restored	[68]
The expression of TIM-3 on NK cells and blasts in the bone marrow of AML patients can be used as a prognosis marker	[69]
Chronic activation of NK cells through the IL-15/mTOR pathway is one of the NK cell exhaustion mechanisms in AML models A similar phenomenon was observed in NK cells derived from AML patients in vitro	[70]
Targeting the PVRIG/PVRL2 axis in AML patients is an efficient immunotherapeutic approach	[71]
NK cells in contact with leukemic cells in the C1498 murine AML model acquire the PD-1 molecule from leukemic cells through the Trogocytosis mechanism in a SLAM receptor-mediated manner	[72]
They showed that the success of leukemia treatment using the anti-PD-L1 antibody in PD-L1^−^ leukemia is due to the targeting of exhausted NK cells that express PD-L1	[73]
Blockade of PD-L1 in murine models of AML prevented the exhaustion of NK cells and increased their anti-leukemic activity	[74]
B7-H3-expressing NK cells increased in the peripheral blood of patients, which was associated with poor prognosis Inhibition of B7-H3 enhanced the cytotoxic activity of NK cells, both in vitro and in vivo	[75]
Blockade of CD155/CD122 on leukemic cells by Flt3 inhibitors increased the cytotoxic activity of NK cells against these cells	[76]
TIGIT was upregulated on NK cells and bone marrow samples derived from AML patients after allogeneic transplantation. This was associated with the downregulation of NK cells in the bone marrow	[77]
TIGIT, CD39, and A2AR checkpoints are essential in inhibiting the activities of NK cells in both peripheral blood and bone marrow Blockade of these checkpoints increased the cytotoxic activity of NK cells	[78]
The expression of TIM-3 in NK cells in newly diagnosed AML patients was associated with a poor prognosis and had prognostic significance	[79]
Newly diagnosed AML patients have a higher frequency of NK cells expressing TIGIT, PD-1, and TIM3 than normal subjects, which was associated with a poor prognosis The expression of these checkpoints was associated with low cytotoxicity, and their inhibition increased the function of NK cells	[80]
TIM-3 expression in NK cells correlates with high functional capacity and better clinical outcomes in AML patients TIGIT was intact, and PD-1 decreased in patients-derived NK cells compared to healthy donors Blockade of TIM-3 (but not TIGIT and PD-1) inhibited the secretion of IFN-γ in NK cells following stimulation	[81]
The use of anti-NKG2A antibodies causes the activation of NK cells and tumor regression in a mouse model of AML	[82]
The interaction of the OX40 molecule on leukemic cells with the OX40L checkpoint causes the activation of NK cells and eliminates leukemic cells in AML patients	[83]
Transposon-engineered CAR-NK cells could efficiently target and destroy CLL-1-expressing AML cell lines, primary AML cells, and even leukemia stem cells	[84]

### Acute lymphocytic leukemia (ALL)

In a study conducted by Duault et al*.* on many B-ALL and T-ALL patients, they suggested that the characterization of NK cells can be used to predict patients' clinical outcomes. They showed that although the cytotoxic function of NK cells in leukemic patients is significantly reduced compared to normal controls, activation markers such as high expression of CD69 and CD56 molecules, production of cytokines, and Calcium signaling are increased. The results also showed that the incomplete maturation of NK cells into effector cells prevents the lysis of leukemic cells by NK cells. They proposed that chronic activation may lead to NK cell exhaustion in ALL patients. The increase of cytokine-producing activated NK cells was associated with disease progression and independently indicated a poor prognosis for ALL [85].

Interestingly, NK cells in the bone marrow and peripheral blood samples of leukemic patients had a similar status. In a study that Bailur and colleagues conducted on bone marrow samples of 35 B-ALL and 26 AML patients, they showed that the activity of NK cells was significantly impaired compared to normal subjects (*n* = 11). While CD16^+^CD57^+^ NK cells were significantly decreased in AML patients, these cells did not change in B-ALL patients. Also, while granzyme secretion and NKG2D expression were reduced in AML patient cells and TIM3 expression was increased, NK cells of B-ALL patients were not different from normal individuals [86]. Moreover, by conducting next-generation sequencing and evaluating gene expression profiles and gene polymorphisms, it has been demonstrated that B-ALL and T-ALL might differentially regulate NK cell exhaustion [87].

In ALL patients, the tumor microenvironment in the bone marrow has been mentioned as one of the influential factors in inducing NK cell exhaustion. Ramírez-Ramírez and colleagues have suggested that molecular CRTAM/Necl-2 interaction in bone marrow niches leads to NK cell exhaustion. They reported that lymphoid progenitor cells in the bone marrow with phenotype CD34^+^CD56^+^CD3^+^CD19^+^CRTAM^+^ are likely to be in the first activation phase and are adjacent to niches with high expression of the ligand nectin-like-2. On the other hand, the bone marrow of ALL patients had a high abundance of CD56^high^CRTAM^+^ NK cells with an exhausted phenotype, which was functionally defective and could produce IL-10 and TGF-β [88].

The phenotypic and functional properties of CD56^−^ NK cells have also been investigated in ALL and chronic myelogenous leukemia (CML) patients treated with dasatinib by Ishiyama and colleagues. This study was conducted on 36 CML or Ph + ALL (Philadelphia chromosome-positive ALL), 26 imatinib or nilotinib‐treated patients, and 15 normal subjects. Their results showed that the frequency of CD56^−^ NK cells was exclusively augmented in dasatinib-treated patients who were cytomegalovirus‐seropositive, and expansion of these cells was accompanied by upregulation of specific NK cells against CMV. The expression of differentiation and activation markers such as CD57, NKG2D, NKG2C, NKp46, NKp30, perforin, and granzyme B was significantly reduced in CD56^−^ NK cells. A comparison of NK cells showed that the characteristics of CD56^−^ and CD56^dim^ cells were relatively similar, but these two cell groups were completely different from CD56^bright^ cells; however, it should be noted that the functional properties of CD56^−^ NK cells were significantly lower than CD56^dim^ cells. Also, inhibition of PD-1 could increase the activity of NK cells, especially CD56^dim^ cells, which was proportional to the expression level of PD-1. This study demonstrated that expansion of CD56^−^PD-1^+^ NK cells indicates chronic activation of NK cells in dasatinib-treated patients who were cytomegalovirus‐seropositive. Further, combination therapy by blockade of the PD-1/PD-L1 axis and dasatinib was suggested as a potential treatment approach for leukemia [89].

A study on leukemic cells in 5 patients with B-cell precursor ALL (BCP-ALL) also showed that microRNA582 inhibits the cytotoxic effects of NK cells on leukemic cells by inducing the CD276 (B7-H3) checkpoint. Therefore, it is suggested that blockade of CD276 or its ligand on NK cells may be a potential therapeutic approach in leukemia [90]. In addition to the discussed inhibitory checkpoints, Rothfelder et al*.*, by studying 44 ALL patients and ALL cell lines (NALM-16, JURKAT, SD-1, REH, SUP -B15, TOM-1, and), have shown that the expression of the OX40L checkpoint on NK cells and its interaction with OX40 on ALL cells causes the activation of NK cells and depletion of Leukemic cells [91].

The use of cytokines that stimulate the activity of NK cells has been proposed as one of the exciting immunotherapy methods that prevent their exhaustion by activating the cytotoxic activity of these cells. Accordingly, a study conducted on a 70Z/3 murine pre-B cell leukemia model (murine model of immune-mediated rejection of the acute lymphoblastic leukemia) showed that treating mice by injecting IL-15-secreting leukemic cells induces and activates NK1.1^+^ cells, accompanied by increased mouse survival time [92].

Few studies have been conducted regarding the exhaustion of NK cells in ALL patients, which causes the lack of accurate conclusions from the results (Table 3). More studies are needed regarding the expression and role of different factors in the exhaustion of NK cells. Studies suggest that the progression of ALL is associated with the exhaustion of NK cells, and targeting exhaustion markers has been associated with promising results; however, there is a need for more comprehensive studies.

**Table 3:** Studies related to the role of exhausted NK cells in ALL

Main findings	Ref
The characterization of NK cells can predict ALL patients' clinical outcomes	[85]
The activity of NK cells in ALL patients was significantly impaired compared to normal subjects	[86]
B-ALL and T-ALL might differentially regulate NK cell exhaustion	[87]
Molecular CRTAM/Necl-2 interaction in bone marrow niches leads to NK cell exhaustion	[88]
Expansion of CD56^−^PD-1^+^ NK cells indicates chronic activation of NK cells in dasatinib-treated patients who were cytomegalovirus‐seropositive Combination therapy by blockade of PD-1/PD-L1 axis and dasatinib was suggested as a potential treatment approach for leukemia	[89]
microRNA582 inhibits the cytotoxic effects of NK cells on leukemic cells by inducing the CD276 (B7-H3) checkpoint in ALL patients	[90]
The expression of the OX40L checkpoint on NK cells and its interaction with OX40 on ALL cells causes the activation of NK cells and depletion of Leukemic cells in ALL patients	[91]
Treating the 70Z/3 murine pre-B cell leukemia model by injecting IL-15-secreting leukemic cells induces and activates NK1.1^+^ cells, accompanied by increased mouse survival time	[92]

## Conclusion

The importance of the anti-cancer function of NK cells in innate immunity has encouraged several investigators to investigate the phenotypic and functional characteristics of these cells in hematopoietic malignancies to evaluate their targeting worth for leukemia immunotherapy.

The phenotype and function of NK cells in almost all leukemia malignancies shifted towards exhausted cells, associated with a poor prognosis. Also, the presence and increase of exhausted NK cells were associated with a poor prognosis. Another point that can be mentioned about exhausted NK cells in leukemic malignancies is the importance of targeting them as a new treatment method. Except for a few exceptions, in almost all studies that targeted the inhibitory checkpoints of exhausted NK cells, all therapeutic studies showed that this treatment method is associated with the recovery of the anti-leukemic function of NK cells and the elimination of leukemic cells.

Regarding the commonality of NK cell exhaustion in different subtypes of leukemia, it seems that leukemia occurrence and progression are associated with the reduced frequency and function of NK cells, hallmarks of the exhaustion process. The upregulation of inhibitory checkpoints and reduced secretion of inflammatory cytokines and toxic mediators are other common NK cell exhaustion characteristics observed in the majority of studies.

Despite the above-indicated deduction, there are unknown issues related to exhausted NK cells in leukemic malignancies, which require comprehensive studies in the future. While most of the studies on exhausted NK cells have been conducted in AML patients, little is known regarding the immunobiology and targeting potential of these cells in other leukemic malignancies, especially CML.

The comparison of exhausted NK cells in the peripheral and bone marrow of leukemia patients is another important issue that should be further studied. In the few studies that have evaluated exhausted NK cells in both peripheral blood and bone marrow, comprehensive information has not been obtained. In some cases, there were even contradictory data. Therefore, extensive studies must investigate these cells in various leukemias in peripheral blood and bone marrow samples. Consideration of disease stage and previously received treatments are also critical issues that should be considered in studies since they can significantly affect the exhaustion process. The defect observed in many studies was that either a proper classification of patients was not provided or patients with different disease stages were not studied simultaneously.

A critical question that future studies should seek to answer is whether the development of cancer leads to the creation of exhausted cells or whether the emergence of exhausted NK cells is one of the reasons for cancer development. Available evidence supports both theories; although these two events may coincide, other factors cause these events. The observation that chemotherapy or anti-leukemia treatments that target the leukemic cells themselves restore the function of NK cells further strengthens the theory that other factors are involved in this issue. Several checkpoints have not yet been investigated in various leukemia diseases, which should be considered in future studies. Also, the molecular mechanisms of NK cell exhaustion have not yet been clearly defined and should be studied further studies.

## Data Availability

No data was used for the preparation of this manuscript.
